# Supplementary material for: Low Genetic Variability in Bemisia tabaci MEAM1 Populations within Farmscapes of Georgia, USA
Source: Insects. 2020 Nov 26;11(12):834. doi: 10.3390/insects11120834 (PMC7760769; doi:10.3390/insects11120834)
Supplement: Supplementary file 1 [file insects-11-00834-s001.pdf]

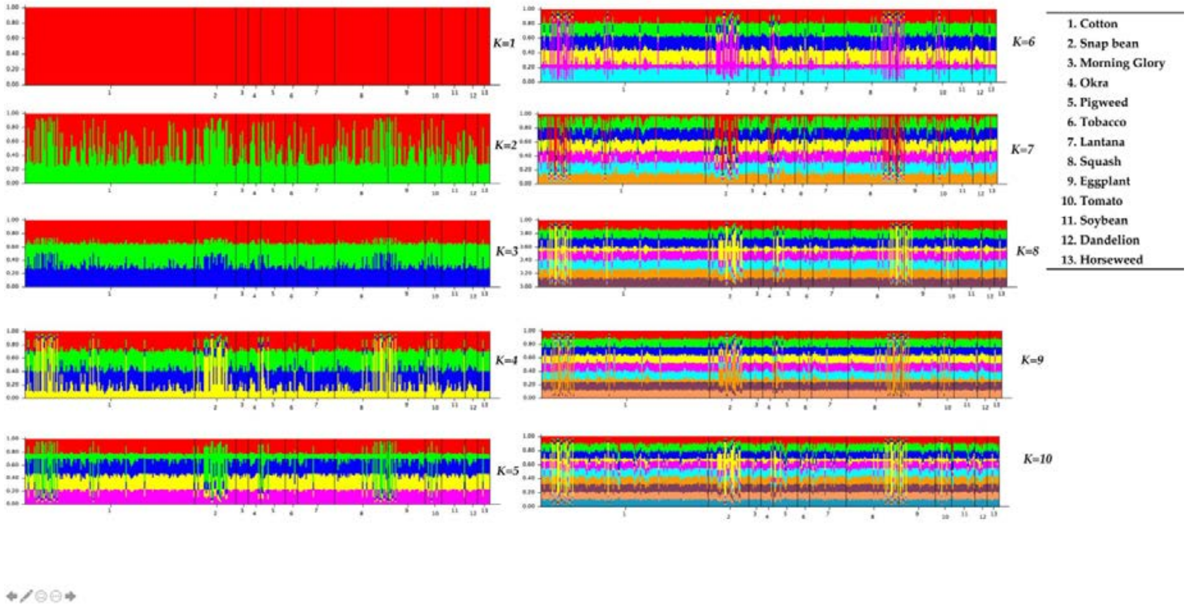

Figure 1. Bayesian clustering analysis results for 35 *B. tabaci* MEAM1 populations based on six microsatellite markers using STRUCTURE v.2.3.2. Structure bar plots at *K* = 1–10.
